# The Virulence-Related MYR1 Protein of *Toxoplasma gondii* as a Novel DNA Vaccine Against Toxoplasmosis in Mice

**DOI:** 10.3389/fmicb.2019.00734

**Published:** 2019-04-09

**Authors:** Bin Zheng, Jianzu Ding, Di Lou, Qunbo Tong, Xunhui Zhuo, Haojie Ding, Qingming Kong, Shaohong Lu

**Affiliations:** ^1^Institute of Parasitic Diseases, Zhejiang Academy of Medical Sciences, Hangzhou, China; ^2^Zhejiang Provincial Institute of Parasitic Diseases, Hangzhou, Zhejiang, China

**Keywords:** *Toxoplasma gondii*, immunization, DNA vaccine, MYR1, Th1/Th2 cytokines

## Abstract

*Toxoplasma gondii* causes serious public health problems, but there is no effective treatment strategy against it currently. DNA vaccines have shown promising findings in this regard. MYR1 is a new virulence factor identified in *T. gondii* that may have potential as a DNA vaccine candidate. We constructed a recombinant eukaryotic plasmid, pVAX1-MYR1, as a DNA vaccine, injected it intramuscularly into BALB/c mice, and evaluated its immunoprotective effects. pVAX1-MYR1 immunization induced a sequential Th1 and Th2 T-cell response, as indicated by high levels of Th1 and mixed Th1/Th2 cytokines at 2 and 6 weeks after immunization, respectively. These findings were corroborated by the antibody assays too. In addition, increased levels of antigen-specific lymphocyte proliferation, CD4^+^ and CD8^+^ T lymphocytes, cytotoxic T lymphocyte activity and cytokine (IFN-γ, IL-12, and IL-10) production were also observed in the immunized mice. These findings showed that pVAX1-MYR1 stimulated humoral and cellular immune responses in the immunized mice. The increased production of IFN-γ and IL-12 was correlated with increased expression of the *T-bet* and *p65* genes of the NF-κB pathway. However, no significant increase was observed in the level of IL-4. The survival of mice immunized with pVAX1-MYR1 was also significantly prolonged compared with the control group mice. Based on all the above findings, the current study proposes that pVAX1-MYR1 can induce a *T. gondii*-specific immune response and should therefore be considered as a promising vaccine candidate against toxoplasmosis. To the best of our knowledge, this is the first report to evaluate the immunoprotective value of an MYR1-based DNA vaccine against *T. gondii*.

## Introduction

*Toxoplasma gondii* is an intracellular protozoa that belongs to the phylum Apicomplexa, which has a global distribution and can cause toxoplasmosis in humans as well as animals (Dubey, 2008). More than one-third of the world’s population has chronic *T. gondii* infection (Pappas et al., 2009). Cats are the only final host of *T. gondii*, and most warm-blooded mammals, including humans, rodents, and birds, are intermediate hosts (Blader et al., 2015). This protozoan can be transmitted through close contact with cats or a pet, eating raw meat, and exposure to soil (Belluco et al., 2016). In humans, toxoplasmosis is usually asymptomatic in individuals with normal immune function. However, in immunocompromised people, such as those with malignant tumors, human immunodeficiency virus-positive individuals and organ transplant recipients, toxoplasmosis may cause serious and progressive complications with poor prognosis or may even lead to death (White et al., 2014). In addition, pregnant women are also at risk of *T. gondii* infection (Hunter and Sibley, 2012). During pregnancy, maternal *T. gondii* infection may have serious consequences such as fetal abortion (Torgerson and Mastroiacovo, 2013).

In general, *T. gondii* tachyzoites actively invade all nucleated cells of the intermediate host, but their replication is ultimately limited by a protective immune response (Sullivan and Jeffers, 2012). A common primary control measure for toxoplasmosis in humans and animals is chemotherapy. Chemotherapy is administered through a combination of pyrimethamine and sulfadiazine, which have a variety of side effects and may cause toxic allergic reactions in and have teratogenic effects on the fetus; further, these two drugs do not prevent the entry of bradyzoites into the tissue cyst (Antczak et al., 2016). As an another treatment modality, a commercial attenuated vaccine (ToxoVax^®^, Intervet B.V.) has been used in the veterinary industry in some areas, but the side effects as well as it high cost have limited the use of this vaccine. Thus, at present, there is no effective control strategy to limit toxoplasmosis in humans and many warm-blooded animals around the world (Li and Zhou, 2018).

Effective and safe anti-*T. gondii* vaccines may be the answer to preventing *T. gondii* infections. In recent years, a large number of studies have been carried out on *T. gondii* vaccines, including attenuated vaccines (Wang J.L. et al., 2017, 2018; Xia et al., 2018), subunit vaccines (Zheng et al., 2013; Ching et al., 2016; Sonaimuthu et al., 2016; Wang S. et al., 2017), exosome vaccines (Beauvillain et al., 2009; Li et al., 2018), DNA vaccines (Zhang et al., 2015; Li and Zhou, 2018) and other types of vaccines (Lee et al., 2018; Zhang N.Z. et al., 2018). Many studies have shown that using antigen-encoding DNA as experimental immunogens can effectively induce humoral and cellular immunity against *T. gondii*. In one of these studies, Zhou et al. reported that the SAG4 DNA vaccine had significant immune responses and improved protection against *T. gondii* (Zhou and Wang, 2017). Further, Zhang Z. et al. (2018) demonstrated that intense cell-mediated and humoral immunity was triggered and defense against *T. gondii* was partially induced after administration of the TgROP21 DNA vaccine. In yet another study, Zheng et al. (2017) found that immunization of mice with pVAX1-TgSPATR can produce humoral and cellular immune responses against *T. gondii* and significantly prolong the survival of mice. Thus, the future of DNA vaccines for the prevention of *T. gondii* infection looks promising.

In recent years, great progress has been made in identifying candidate vaccines for *T. gondii* infection that can induce a protective immune response. Of these potential vaccine antigens, *T. gondii* Myc regulation 1 (MYR1) seems to be particularly promising. MYR1 is a new virulence factor identified in *T. gondii*. It is a secreted protein that is ultimately localized within the parasitophorous vacuole and at the parasitophorous vacuole membrane, and it plays a key role in infected host cells. During *T. gondii* infection, MYR1 can upregulate the expression of c-Myc in host cells, mediating the interaction between the host and host cells, for example, by affecting the host cell cycle. In addition, the MYR1 protein is required for *T. gondii* tachyzoites to regulate several other important signaling pathways in the host, including those mediated by the dense particle effectors GRA16 and GRA24. MYR1 is also important for the transfer of *T. gondii* effector molecules from parasite vacuoles to the host cytoplasm or nucleus. In a mouse infection model, the virulence of MYR1-knockout *T. gondii* was found to be severely attenuated, and it did not result in death of the mice (Franco et al., 2016). Moreover, we have used some bioinformatics software to predict that MYR1 show good B-cell and T-cell epitopes (Zhou et al., 2016). These findings indicate that MYR1 may be a great potential vaccine candidate, but no studies have explored this possibility.

The aim of this study was to evaluate the potential of MYR1 as a candidate vaccine against *T. gondii* infection in mice. We constructed an MYR1 eukaryotic plasmid and intramuscularly administered it to BALB/c mice to evaluate the immunoprotective effect of this DNA vaccine on infection of the BALB/c mouse model with the highly virulent *T. gondii* RH strain.

## Materials and Methods

### Ethics Statement

This study was carried out in strict accordance with the recommendations of the Guide for the Care and Use of Laboratory Animals according to the Animal Ethics Procedures and Guidelines of the People’s Republic of China. The protocol was approved by the Institutional Animal Care and Use Committee (IACUC) of Zhejiang Academy of Medical Sciences.

### Experimental Mice, Parasites and Preparation of Soluble Tachyzoite Antigen

Six-week-old BALB/c mice were purchased from the Experimental Animal Center of Zhejiang Academy of Medical Sciences, China. All the mice were maintained under specific-pathogen-free standard conditions and the experimental procedures were in accordance with Chinese legislation on the use and care of laboratory animals. *T. gondii* tachyzoites of the virulent wild-type RH strain (type I) were maintained in our laboratory through serial passage in human foreskin fibroblasts grown in Dulbecco’s modified Eagle’s medium (Gibco, Carlsbad, CA, United States) supplemented with 5% fetal bovine serum (Gibco, Carlsbad, CA, United States) in a humidified chamber containing 5% CO_2_ at 37°C. Soluble tachyzoite antigen was prepared from *T. gondii* tachyzoites as described previously (Wang et al., 2013).

### Cloning and Molecular Characterization of MYR1

Total RNA from the tachyzoites of the *T. gondii* RH strain was extracted by Trizol reagent (Invitrogen, Carlsbad, CA, United States) according to the manufacturer’s protocol, and the cDNA was constructed. The entire coding sequence of the *T. gondii* MYR1 (TGGT1_254470) gene was amplified by PCR with synthetic primers for MYR1: forward primer, 5′-GCCGATATCATGCCGCCACAGAATCGTAACG-3′; reverse primer, 5′-GCCGCGGCCGCTCACGAATTATGTGACTGAC-3′ (the introduced *Eco*RV and *Not*I recognition sites are underlined). The PCR product of the MYR1 gene was subcloned into the pMD19-T vector (Takara, Dalian, China). MYR1-positive clones were selected for sequencing.

### Preparation and Transfection of the pVAX1-MYR1 Plasmid

The MYR1 gene was digested with the restriction enzymes corresponding to the *Eco*RV and *Not*I sites, and purified from agarose gels. The MYR1 gene fragment was inserted into the eukaryocyte vector pVAX1, which was then named pVAX1-MYR1. The recombinant plasmids were then transformed into *Escherichia coli* DH5a, and the transformation was confirmed by sequencing. pVAX1-MYR1 was purified using an Endofree plasmid giga kit (Qiagen, Chatsworth, CA, United States). The DNA concentration was determined by absorbance at 260 nm using NanoDrop 2000. Finally, the recombinant plasmid was diluted to a concentration of 1 mg/ml with sterile endotoxin-free phosphate-buffered saline (PBS) and stored at −20°C until use. pVAX1-MYR1 expression was analyzed by transfection of HEK293 cells with the Lipo2000 reagent (Invitrogen, Carlsbad, CA, United States), according to the manufacturer’s instructions. After 48 h, cell monolayers and supernatants were collected and stored at −20°C. Expression of MYR1 in the transfected cells was then analyzed by real-time PCR (RT-PCR), western blotting and indirect immunofluorescence assay.

### Preparation of the rMYR1 Protein and Production of Polyclonal Antibodies Against rMYR1

The pET28a-MYR1 plasmid was constructed using the same method used to construct the pVAX1-MYR1 plasmid. This pET28a-MYR1 plasmid was transformed into *E. coli* BL21(DE3) and induced to express the rMYR1 protein *in vitro* using Isopropyl β-D-Thiogalactoside. The rMYR1 protein was purified by affinity chromatography using Ni^2+^-NTA agarose columns (Qiagen, Hilden, Germany). The purity of the eluted proteins was analyzed by 12% sodium dodecyl sulfate-polyacrylamide gel electrophoresis and Coomassie blue staining. rMYR1 was dialyzed against PBS (pH 7.2), filtered throughout a 0.22-μm pore membrane and stored at −70°C until use. To generate polyclonal antibodies against rMYR1, New Zealand experimental rabbits were immunized subcutaneously with 200 μg of the rMYR1 protein emulsified with an equal volume of Freund’s complete adjuvant (Sigma-Aldrich, United Kingdom). Two weeks later, booster immunization was performed after emulsification with 200 μg rMYR1 protein and an equal volume of incomplete Freund’s adjuvant (Sigma-Aldrich, United Kingdom). A second booster immunization was performed after 2 weeks. Two weeks after the second booster immunization, the rabbits were sacrificed and serum was extracted.

### Immunization Schedule and Challenge

Four groups of female BALB/c mice (25 mice per group) were inoculated three times at 2-week intervals through a single subcutaneous injection: Group I received no treatment and served as a healthy control group; Group II was administered 100 μL of sterile PBS and served as the negative control group; Group III was administered 100 μL of PBS containing 100 μg of the empty pVAX1 (vector control group); and Group IV was administered 100 μL of sterile PBS containing 100 μg pVAX1-MYR1. Blood samples from each group of mice were collected before immunization and on days 13, 27, and 41 after first immunization, and serum was stored at −20°C.

Two weeks after the final inoculation, ten mice each from the experimental group and the control group were sacrificed, and their spleens were collected for lymphocyte proliferation assay, cytokine measurement, analysis of T cell subsets and cytotoxic T lymphocyte (CTL) activity assay. Further, 100 tachyzoites were obtained from the highly virulent *T. gondii* RH strains and were used to intraperitoneally challenge ten immunized mice per group 3 weeks after the final inoculation. Survival was monitored daily.

### Post-vaccination Antibody Measurement

Indirect ELISA was used to determine mouse serum IgG, IgG1, and IgG2a antibody levels. Briefly, 96-well microtiter plates were coated overnight at 4°C with rMYR1 (1 μg/well). These plates were then washed three times with PBS containing 0.05% Tween-20. Non-specific binding sites were blocked with PBS containing 10% bovine serum albumin and incubated with mouse sera diluted 1:100 with PBS for 1 h at 37°C. HRP-conjugated goat anti-mouse IgG, IgG1, and IgG2a (Abcam, Cambridge, United Kingdom) diluted to 1:10,000 were used to detect the bound antibodies. Immune complexes were revealed with 3,3′,5,5′-Tetramethylbenzidine dihydrochloride as the substrate. An automatic ELISA reader (BioTek, United States) was utilized to measure the absorbance values at 450 nm, and all the tests were run in triplicate.

### Lymphocyte Proliferation Assay

Two weeks after the final booster injection, spleens were harvested from five mice in each group. Splenocyte suspensions were prepared and cultured in 96-well plates in triplicate at a density of 2 × 10^5^ cells/well in Dulbecco’s modified Eagle’s medium supplemented with fetal bovine serum. Thereafter, the cultures were stimulated with either 10 μg/ml rMYR1 or 5 μg/ml concanavalin A (ConA) (Sigma, St. Louis, MO, United States). According to the manual, the plates were incubated in 5% CO_2_ at 37°C for 68 h, after which 50 μl of CCK-8 solution (Dojindo, Japan) was added to each well and incubated for 4 h. When the incubation was completed, 200 μl of dimethyl sulfoxide was added to each well before all the contents were discarded, and absorbance was measured at 450 nm with an ELISA reader. The results were expressed as the stimulation index (SI) and were calculated as follows: (OD570 rMYR1/OD570 Control)/(OD570 ConA/OD570 Control).

### Cytokine and Cytokine-Related Transcription Factor Assay

As described for the lymphocyte proliferation assay, splenocytes harvested from each group were cultured with rMYR1 or medium alone (negative control) in 96-well microtiter plates. According to the manufacturer’s instructions and published methods (Wang J.L. et al., 2017), cell-free supernatant was collected and assessed for the level of IL-4 at 24 h, IL-10 at 72 h, and IFN-γ and IL-12 at 96 h, using commercial ELISA kits (eBioscience, United States). The analysis was performed through three independent experiments.

The expression of the transcription factors p65 and T-bet of the NF-κB pathway was detected by RT-PCR and western blotting, in order to determine their roles in mediating the increased production of T-cell cytokines (e.g., IFN-γ and IL-12). The SYBR Green qPCR Mix was purchased from Bio-Rad (United States). RT-PCR was performed on CFX96 Touch (Bio-Rad). The amplification reactions were performed under the following conditions: 50°C 2 min, 95°C 2 min, 40 cycles of 95°C for 15 s, and 60°C for 30 s. Melting curve analysis was carried out under the following conditions: 1 min at 95°C, 65°C for 2 min, and progressive increase from 65 to 95°C to ensure that a single product was amplified in each reaction. All measurements were run in triplicate. The primers for amplification of p65 and T-bet genes are listed in Table 1.

**Table 1:** RT-PCR primers used to amplify the *NF-κB p65, T-bet*, and *β-actin* genes designed by DNASTAR software.

Primer name	Sequence
NF-κB p65-F	5′-GAACCAGGGTGTGTCCATGT-3′
NF-κB p65-R	5′-TCCGCAATGGAGGAGAAGTC-3′
T-bet-F	5′-GCCAGGGAACCGCTTATATG-3′
T-bet-R	5′-TGGAGAGACTGCAGGACGAT-3′
β-Actin-F	5′-GCTTCTAGGCGGACTGTTAC-3′
β-Actin-R	5′-CCATGCCAATGTTGTCTCTT-3′

### Flow Cytometry Analysis of T-cell Subsets

Flow cytometry was used to analyze the percentage of T-cell subsets CD4^+^ and CD8^+^ in the splenocytes of mice from the four groups (pVAX1-rMYR1, pVAX1, PBS, and healthy control). Splenocyte suspensions (5 × 10^5^ cells/ml) were dually stained with anti-mouse CD3e-FITC + anti-mouse CD8-PE and anti-mouse CD3e-FITC + anti-mouse CD4-PE (eBioscience, United States) for 30 min at 4°C in the dark. Cell population analysis was conducted with the FACScan flow cytometer using the CellQuest software (BD Biosciences, Franklin Lakes, NJ, United States).

### CTL Activity Assays

Mouse spleen lymphocytes were obtained and prepared as described in the lymphocyte proliferation experiment. CTL activity was measured using CytoTox96^®^ Non-Radioactive Cytotoxicity Assay Kits (Promega, United States). Briefly, spleen cell cultures were co-cultured with 100 U/ml recombinant murine IL-12 (eBioscience, United States) and used as effector cells. Five days later, mouse Sp2/0 cells were transfected with pVAX1-MYR1 using LipofectamineTM 2000 reagent (Invitrogen, United States) according to the manufacturer’s instructions and used as target cells. The effector cells were mixed with the target cells at a ratio of 10:1, 20:1, 40:1, 80:1, and incubated for 6 h in a humidified chamber at 37°C, 5% CO_2_. The percentage of specific lysis is calculated as follows: (Experimental – Effector Spontaneous – Target Spontaneous)/(Target Maximum – Target Spontaneous) × 100.

### Statistical Analysis

Survival time was analyzed using the Kaplan–Meier method. Differences in the levels of antibodies, lymphocyte proliferation and cytokines between groups were evaluated using independent-sample *t*-test, with the SSPS statistical analysis software (SPSS Inc., Chicago, IL, United States). *p*-values that were <0.05 were considered to indicate statistical significance.

## Results

### Expression of rMYR1 in *E. coli* BL21 (DE3) and MYR1 in HEK293 Cells

**FIGURE 1:**
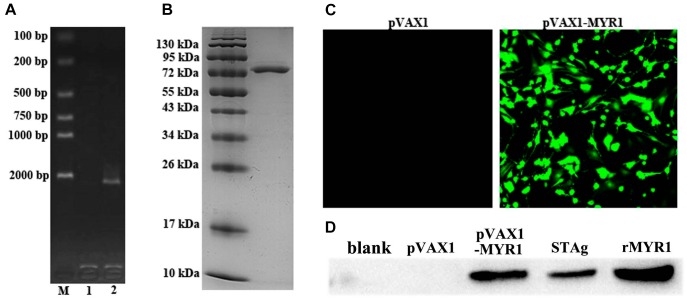
Expression of pET-28a-MYR1 in *E. coli* BL21 (DE3) and pVAX1-MYR1 in HEK293 cells. **(A)** Agarose gel analysis of the PCR-amplified *myr1* gene. M: Marker, 1: Blank control, 2: *T. gondii* CDS. **(B)** SDS-PAGE of recombinant MYR1 protein after purification by Ni-NTA. **(C)** Immunofluorescence analysis of the expression of pVAX1-MYR1 in HEK293 cells. **(D)** Western blotting was used to detect the expression of MYR1 in HEK293 cells and STAg. Blank, non-transfected cells; pVAX1, empty pVAX1 transfected cells; pVAX1-MYR1, pVAX1-MYR1-transfected cells; STAg, soluble antigen of *T. gondii*; rMYR1, positive control.

The coding sequence of MYR1 was 2199 bp in size and encoded a 733-amino acid protein with a predicted molecular weight of 80.57 kDa (Figure 1A). Most of the recombinant MYR1 was expressed in *E. coli* as a soluble fusion protein with His-tag when bacterial growth occurred at 37°C. Soluble rMYR1 was purified by Ni affinity chromatography. rMYR1 had a molecular weight of approximately 80 kDa, as determined by SDS-PAGE (Figure 1B). The rabbit polyclonal antibody prepared using rMYR1 specifically recognized native MYR1 in the soluble antigen of *T. gondii* (Figure 1D).

We evaluated *in vitro* expression of pVAX1-MYR1 by immunofluorescence assay at 48 h after transfection of HEK293 cells. Cells transfected with pVAX1-MYR1 showed specific green fluorescence, whereas cells transfected with empty pVAX1 did not display cellular immunofluorescence (Figure 1C). The western blotting results revealed a single band (∼80 kDa) of the expected molecular size, indicating that pVAX1-MYR1 was expressed in HEK293 cells (Figure 1D). No protein band was detected in cells transfected with the empty pVAX1 vector and non-transfected cells. This finding indicates that the MYR1 protein was expressed by pVAX1-MYR1 in HEK293 cells.

### Specific IgG and IgG Isotypes Induced by pVAX1-MYR1 Immunization

**FIGURE 2:**
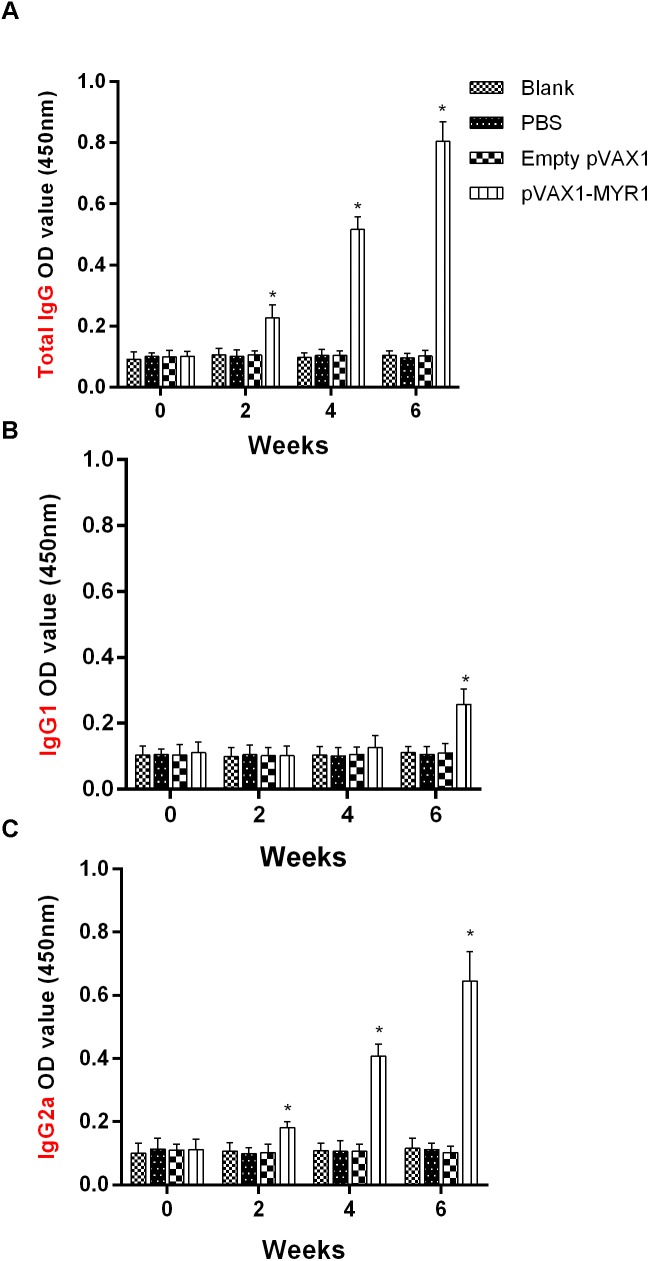
Humoral response in BALB/c mice induced by pVAX1-MYR1 immunization. **(A)** Total IgG, **(B)** IgG1, and **(C)** IgG2a. The total IgG and IgG subclass (IgG1 and IgG2a) titers were determined at 0, 2, 4, and 6 weeks after first vaccination. Data are expressed as the mean ± SD (*n* = 9) values from three independent experiments. ^∗^*p* < 0.05, indicates a significant difference in comparison with the control groups.

In order to detect the levels of anti-*T. gondii* MYR1 antibodies, we tested sera samples from all the groups with ELISA. As shown in Figure 2A, anti-MYR1 antibody was detectable as early as 2 weeks after first inoculation in the pVAX1-MYR1 group: the OD450 value of total IgG antibody was 0.82 ± 0.03 (mean ± SD) at 2 weeks after the last immunization. IgG levels were significantly higher in the serum of mice immunized with pVAX1-MYR1 than in the serum of the control group mice (*p* < 0.05), and the antibody levels increased with continuous immunization. As expected, no increase in antibody levels was detected in any of three control groups (Figure 2A).

The distribution of the IgG subclasses IgG1 and IgG2a was analyzed in the serum of mice immunized with pVAX1-MYR1 using rMYR1 as the coating antigen. Compared to non-vaccinated mice, the level of IgG2a was significantly higher in vaccinated mice at 2, 4, and 6 weeks after first inoculation (*p* < 0.05) (Figure 2B). In contrast, the level of IgG1 only increased in the vaccinated mice at 6 weeks after vaccination (*p* < 0.05), as compared with the non-vaccinated mice (Figure 2C). These results indicate that vaccination with pVAX1-MYR1 in mice elicits a Th1-type immune response at 2 weeks after vaccination and a mixed Th1/Th2 immune response at 6 weeks after vaccination.

### Stimulation of Spleen Lymphocyte Proliferation and IFN-γ, IL-12, and IL-10, but Not IL-4, Production by Spleen Lymphocytes by pVAX1-MYR1 Immunization

The lymphocyte proliferation assay was carried out to analyze the proliferation of splenocytes stimulated with rMYR1 or ConA at 2 weeks after the last immunization. A significantly higher SI value was obtained in the pVAX1-MYR1-immunized groups compared with the control groups (*p* < 0.05) (Table 2). There was no statistically significant difference between the three control groups (*p* > 0.05). These results showed that the lymphocytes of the vaccinated mice were successfully stimulated.

**Table 2:** Cytokine production and splenocyte proliferation in BALB/c mice immunized with pVAX1-MYR1.

Groups (*n* = 5)	Cytokine production (pg/ml)				Proliferation (SI)^a^
	IFN-γ	IL-12	IL-4	IL-10	
Blank control	35.23 ± 3.28	29.22 ± 2.99	30.01 ± 5.13	20.34 ± 1.98	0.87 ± 0.15
PBS	38.23 ± 4.35	32.08 ± 3.19	31.29 ± 4.29	19.56 ± 2.91	0.78 ± 0.09
pVAX1	36.21 ± 3.98	35.01 ± 3.78	33.45 ± 3.68	21.55 ± 2.89	0.81 ± 0.12
pVAX1-MYR1	839.87 ± 60.39^∗^	405.29 ± 67.81^∗^	31.26 ± 3.28^∗^	86.81 ± 9.03^∗^	2.72 ± 0.28^∗^

*^a^SI stands for stimulation index. Splenocytes from mice were harvested 2 weeks after the last immunization. Data are expressed as the mean ± SD values (n = 5) from three independent experiments. ^∗^p < 0.05, indicates a significant difference in comparison with the control groups.*

In order to determine whether pVAX1-MYR1 immunization enhances the Th1 or Th2 cytokine response, IFN-γ, IL-12, IL-4, and IL-10 levels in the supernatants of antigen-stimulated splenic cells were determined (Table 2). Compared to the control groups, significantly higher levels of IFN-γ and IL-12 were produced in the supernatants of restimulated splenocyte cultures from mice immunized with pVAX1-MYR1 (*p* < 0.05). IFN-γ and IL-12 are Th1-associated cytokines that are critical for protection against *T. gondii*. The concentration of IFN-γ and IL-12 in the pVAX1-MYR1-immunized BALB/c mice was 839.87 ± 60.39 pg/ml and 405.29 ± 67.81 pg/ml, respectively. In addition, mice immunized with pVAX1-MYR1 also showed significantly higher levels of IL-10 (*p* < 0.05). However, in the pVAX1-MYR1-immunized group, the concentration of IL-4 did not increase compared to the control groups (*p* > 0.05). These findings confirm the results for the anti-*T. gondii* IgG isotype experiment, which showed that immunization with pVAX1-MYR1 induces a mixed Th1/Th2 response.

### Increase in CD4^+^CD8^−^ and CD8^+^CD4^−^ T-cell Levels After pVAX1-MYR1 Immunization

We further characterized the T-cell subsets by flow cytometry to detect the number of CD3^+^CD4^+^CD8^−^ and CD3^+^CD8^+^CD4^−^ T cells in the spleens of mice from each group (Table 3). In the mice immunized with pVAX1-MYR1, the levels of CD3^+^CD4^+^CD8^−^ T cells was 25.64 ± 3.19% and the levels of CD3^+^CD8^+^CD4^−^ T cells was 11.23 ± 1.15%; these amounts were significantly higher than those in the control groups (*p* < 0.05). As expected, the proportion of these two T-cell subtypes was not significantly different between the three control groups (*p* > 0.05). These findings indicate that the frequency of CD4^+^ and CD8^+^ T cells was augmented after pVAX1-MYR1 immunization.

**Table 3:** Percentage of CD4^+^ T cells and CD8^+^ T-cell subsets in immunized BALB/c mice.

Groups	CD3^+^ CD4^+^ CD8^−^ (%)	CD3^+^ CD8^+^ CD4^−^ (%)
Blank control	9.34 ± 1.91	3.75 ± 0.38
PBS	9.12 ± 2.14	4.01 ± 0.41
pVAX1	10.23 ± 2.11	3.66 ± 0.43
pVAX1-MYR1	25.64 ± 3.19^∗^	11.23 ± 1.15^∗^

*Splenocyte suspensions were analyzed by flow cytometry. Data are expressed as the mean ± SD values (n = 5) from three independent experiments. ^∗^p < 0.05, indicates a significant difference in comparison with the control groups.*

### Increased Expression of p65 and T-bet in Spleen Lymphocytes After pVAX1-MYR1 Immunization

**FIGURE 3:**
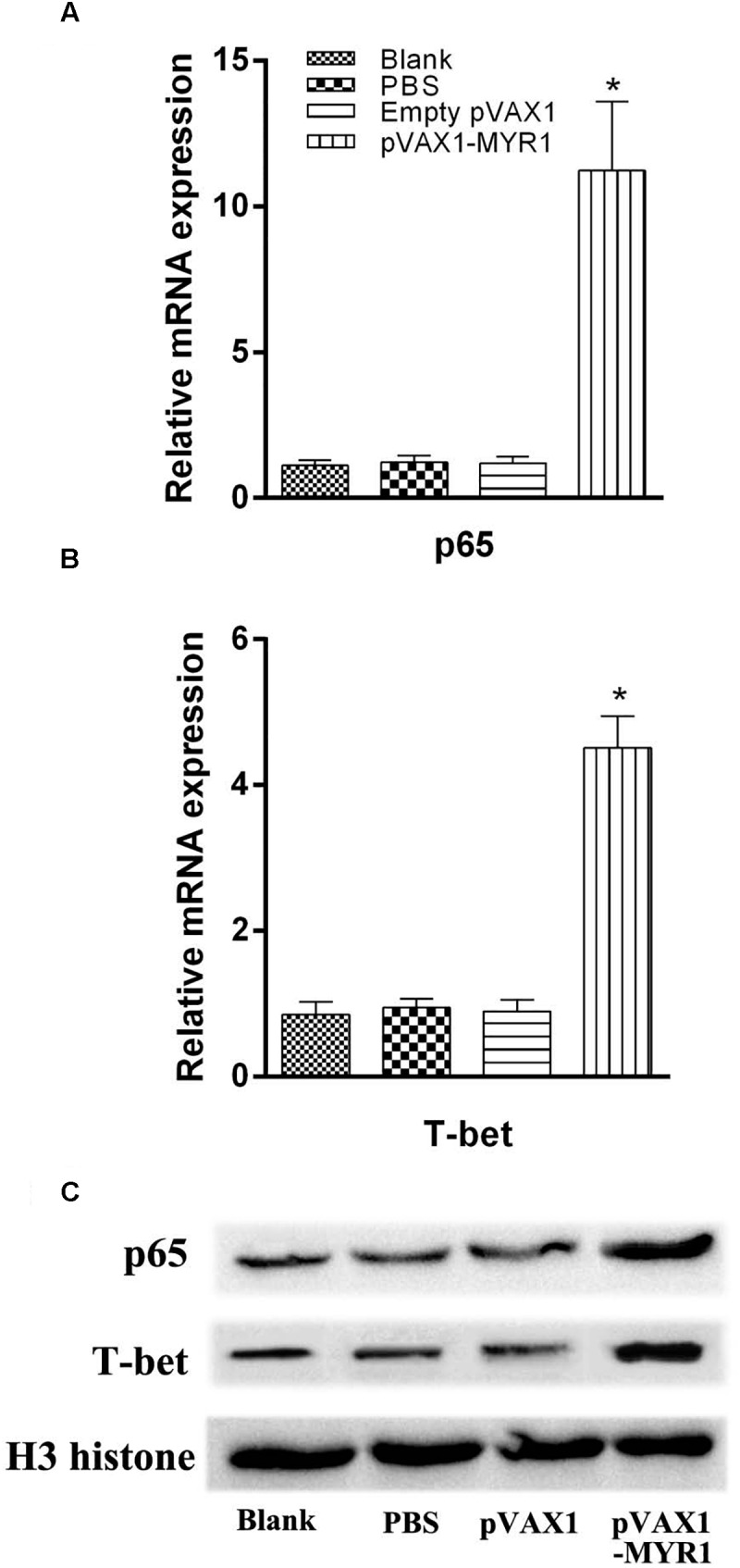
Expression of the transcription factors T-bet and p65. The mRNA and protein expression levels of p65 and T-bet. **(A,B)** The mRNA levels of p65 and T-bet; **(C)** The protein levels of p65 and T-bet. Data are expressed as the mean ± SD values (*n* = 5) from three independent experiments. ^∗^*p* < 0.05, indicates a significant difference in comparison with the control groups.

Expression of the transcription factors T-bet and p65 was detected using RT-PCR and western blotting. We examined the difference in the mRNA level and nuclear protein expression of these two genes between control mice and pVAX1-MYR1-immunized mice. We found that the expression of both T-bet and p65 was significantly higher in pVAX1-MYR1-immunized mice than in the control group (*p* < 0.05) (Figure 3). These results indicate that T-bet and p65 are induced by pVAX1-MYR1 immunization and are likely to increase the production of IFN-γ and IL-12.

### Increase in CTL Activity After pVAX1-MYR1 Immunization

**FIGURE 4:**
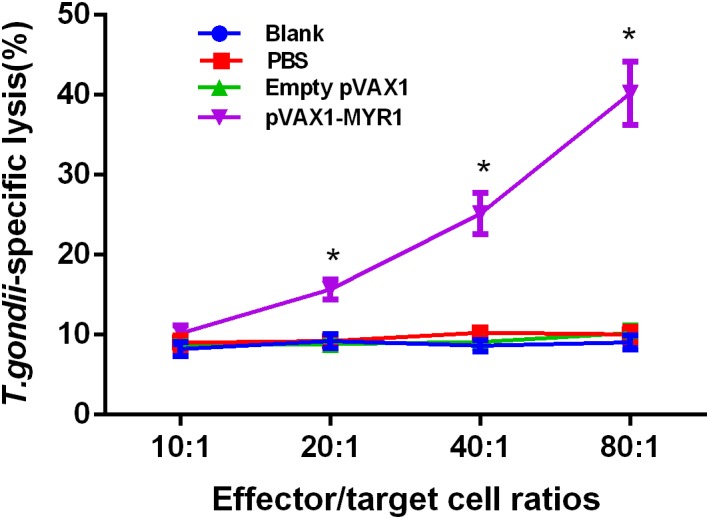
CTL activities of spleen lymphocytes in pVAX1-MYR1-immunized mice. The effector-to-target cell ratios are indicated along the horizontal axis. The vertical axis shows the percentage of *T. gondii*-specific lysis. Data are expressed as the mean ± SD values (*n* = 5) from three independent experiments. ^∗^*p* < 0.05, indicates a significant difference in comparison with the control groups.

We found that as the ratio of effector cells to target cells gradually increased, CTL activity was gradually enhanced. The highest cytotoxic activity was observed at an effector cell/target cell ratio of 80:1 (Figure 4), and it was significantly higher than that of the control groups (*p* < 0.05). In addition, CTL activities were not significantly different between the three control groups (*p* > 0.05).

### Improved Survival After *T. gondii* Infection in pVAX1-MYR1-Immunized Mice

**FIGURE 5:**
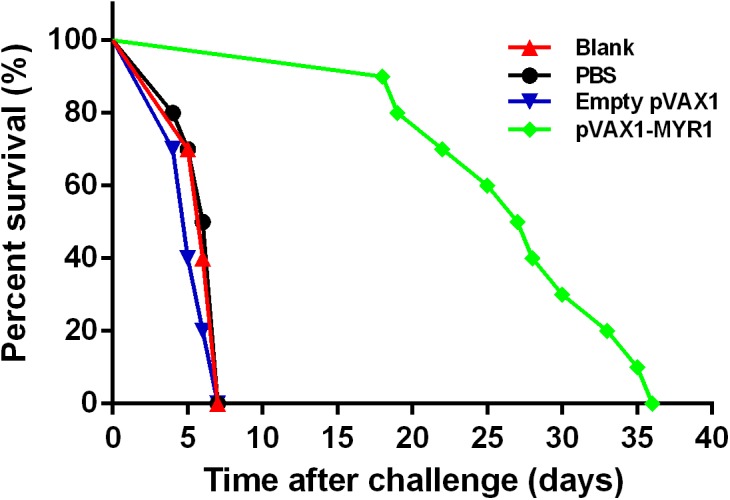
Survival curves of BALB/c mice after challenge. Each group comprised 10 mice. Survival was significantly higher in the pVAX1-MYR1-immunized mice than in the control mice. ^∗^*p* < 0.05, indicates a significant difference in comparison with the control groups.

We challenged mice from all the groups with tachyzoites of the highly virulent *T. gondii* strain RH 3 weeks after inoculation and observed the survival time (Figure 5). No differences were observed between the three control groups with regard to the mean survival time at 6 days after challenge (*p* > 0.05). A significant increase in survival time was observed in the group immunized with pVAX1-MYR1 compared with the control groups. The average survival time of the pVAX1-MYR1 immunization group was 27.3 ± 6.3 days, while the control group mice died by the 7th day after infection.

## Discussion

In the present study, we investigated the possibility of using the novel virulence-related *T. gondii* protein MYR1 as a candidate DNA vaccine to prevent animal toxoplasmosis. We used pVAX1 as a DNA vaccine vector which is consistent with the Food and Drug Administration (FDA) document. The results showed that immunization of mice with pVAX1-MYR stimulated efficiently prolonged protective immunity against the highly virulent RH strain of *T. gondii*. The humoral response is thought to be critical for the immunity of *T. gondii* by inhibition of the adsorption on cells and promoting macrophage killing of intracellular parasites (Yoshida et al., 2017). In the present study, mice immunized with pVAX1-MYR1 gradually produced high levels of *T. gondii*-specific IgG at 2, 4, and 6 weeks after immunization. This antibody plays an important role in preventing subsequent *T. gondii* infection and controlling cyst reactivation during chronic infection (Lakhrif et al., 2018). Many studies have demonstrated that a Th1-biased response is required for effective protection against naturally occurring *T. gondii* infections (Yarovinsky, 2014). We therefore determined the antibody types triggered by pVAX1-MYR1 immunization by assaying the antibody subclasses IgG1 and IgG2a. pVAX1-MYR1-immunized mice displayed IgG2a as the primary antibody class during early infection; later, upregulation of IgG1 was observed in the chronic phase, possibly as a result of the conversion of Th1 to Th2. Two weeks after immunization, we observed high IgG2a antibody titers, which indicate induction of a Th1 immune response. However, the IgG1 antibody levels only increased 6 weeks after immunization, indicating induction of a mixed Th1/2 immune response. These results demonstrate that the anti-*T. gondii* IgG2a and IgG1 antibodies are upregulated in sequence. These results are confirmed by the results of the cytokine assay conducted on spleen cell culture supernatants, in which the levels of Th1 cytokines (IFN-γ and IL-12) and Th2 cytokines (IL-10) in mice immunized with pVAX1-MYR1 were significantly higher than those in the control mice; however, IL-4 production was not induced. Altogether, our findings indicate that IgG2a is triggered by IFN-γ in mice, and that a bias in Th1 response is associated with early IFN-γ production. These results are similar to those of previous research (Wang J.L. et al., 2017, 2018). Our results thus indicate that immunization with pVAX1-MYR1 promotes both cell (Th1)- and humoral (Th2)-mediated immune responses in BABL/c mice.

Increased production of Th1 cytokines is a key mechanism for an effective immune response against *T. gondii* infection (Pifer and Yarovinsky, 2011). During natural *T. gondii* invasion, the level of IFN-γ determines the fate of the infection. IFN-γ can inhibit the replication of *T. gondii* in infected cells through various mechanisms, including induction of the inhibitory protein guanamine 2,3-dioxygenase (IDO), inducible nitric oxide synthase (iNOS) and the effector proteins immunity-related GTPases (IRGs) and guanylate-binding proteins (GBPs). IDO depletes tryptophan, which is necessary for the growth of *T. gondii*. iNOS is capable of producing the highly toxic metabolite nitric oxide and consuming arginine, which is also necessary for the growth of *T. gondii*, to restrict parasite replication. IRGs and GBPs can destroy the parasitophorous vacuole, which once damaged leads to the elimination of *T. gondii* from the cytoplasm of the infected cells (Sturge and Yarovinsky, 2014; Yarovinsky, 2014). In the present study, *in vitro* re-stimulation of spleen lymphocytes with rMYR1 resulted in a significant increase in IFN-γ and IL-12 production in pVAX1-MYR1-immunized mice and also improved survival. These results indicate that the pVAX1-MYR1 vaccine can trigger increased IFN-γ and IL-12 production via the Th1-type immune response and help to effectively prevent acute *T. gondii* infection. In our study, an increase in IL-10 levels was also observed in pVAX1-MYR1-immunized mice. IL-10 functions as a regulatory cytokine that is very important for the prevention of immunopathological damage caused by high levels of IFN-γ as part of the Th1 response. The Th1/Th2 cytokine balance in the pVAX1-MYR1-immunized mice was adequate to control the dissemination of tachyzoites, but not excessive enough to elicit significant inflammation. Thus, the high IL-10 levels contributed to longer survival time in the pVAX1-MYR1-immunized mice (Wang J.L. et al., 2017, 2018). However, the levels of IL-4 in pVAX1-MYR1-immunized mice was similar to those in the controls; this means that immunization with this DNA vaccines did not promote sufficient B cell proliferation and mast cell responses (Zhou and Wang, 2017). This finding also explains why the pVAX1-MYR1 vaccine did not provide complete protection from acute *T. gondii* infection. In the present study, we observed increased IL-12 levels in the immunized mice. IL-12 is important for IFN-γ production, which in turn induces the differentiation of Th1 T lymphocytes and possibly CD8^+^ and NK cells to control *T. gondii* infection. It was reported that inhibition of IL-12 expression resulted in 100% mortality in mice infected with *T. gondii* (Morgado et al., 2014). The NF-κB pathway plays a role in inducing IL-12 secretion in the response to toxoplasmosis (Jensen et al., 2011). In the present study, a significant increase in the expression level of the transcription factor p65 of the NF-κB pathway was observed in pVAX1-MYR1-immunized mice compared to mice in the control group. This indicates activation of the NF-κB pathway as an additional mechanism for increased IFN-γ production in immunized mice. In addition, the expression level of the transcription factor T-bet was also significantly increased in the pVAX1-MYR1-immunized mice. This indicates that increased T-bet-mediated activation of CD4^+^ T cells and natural killer cells may also play a role in increased IFN-γ production (Zhu et al., 2012).

Given the intracellular localization of *T. gondii*, specific T lymphocyte activation (CD4^+^ T cells and CD8^+^ T cells) may play an important role in controlling the spread and development of *T. gondii* infection. CD8^+^ T cell-mediated CTL activity is particularly important in this regard (Kemball et al., 2007; Dupont et al., 2012). In the present study, we found that a significantly higher level of splenocyte proliferation was induced by pVAX1-MYR1 immunization; this indicates that an activated cellular immune response was induced in the immunized mice. In addition, the levels of both CD4^+^ and CD8^+^ T cells were also significantly increased in the immunized mice. Thus, all these immune cells may play a synergistic role in the response against *T. gondii*, and may also be responsible for the increased secretion of IFN-γ. Moreover, *T. gondii*-specific CD8^+^ CTL activity plays an important role in controlling *T. gondii* replication and clearance. Therefore, *T. gondii*-specific CTL induction is an important strategy for the development of effective *T. gondii* vaccines. However, in previous studies on *T. gondii* vaccines, detection of CTL activity was rarely found. In the present study, we found that mouse spleen cells immunized with pVAX1-MYR1 and stimulated again with rMYR1 were able to induce high levels of CTL activity. Taken together, the results show that the pVAX1-MYR1 DNA vaccine can induce strong humoral and cellular immune responses against *T. gondii*.

The survival rate of vaccinated mice against *T. gondii* challenge is considered as the most direct indicator of the effectiveness of a candidate vaccine. Therefore, 3 weeks after the last immunization with pVAX1-MYR1, we conducted a survival assessment by infecting the vaccinated mice intraperitoneally with tachyzoites from the highly virulent *T. gondii* RH strain. Although the mice did not survive after the infection, survival time was significantly and efficiently increased in the mice immunized with pVAX1-MYR1. Thus, the pVAX1-MYR1 vaccine may have potential for preventing *T. gondii* infection. In future experiments, it would be helpful to evaluate the efficacy of pVAX1-MYR1 immunization by comparing the brain tissue cyst burden in vaccinated and control groups by using low virulence strains of *T. gondii* and analyzing the potential immune response mechanisms involved.

## Conclusion

We have analyzed the efficacy of a new *T. gondii* DNA vaccine, pVAX1-MYR1, which expresses a novel virulence-related *T. gondii* protein, MYR1. Our results indicate that intramuscular immunization of BABL/c mice with pVAX1-MYR1 can generate an immunoprotective response against *T. gondii* infection and also increase survival time. Thus, pVAX1-MYR1 may have potential as a promising candidate vaccine against *T. gondii* infection.

## Ethics Statement

This study was carried out in strict accordance with the recommendations of the Guide for the Care and Use of Laboratory Animals according to the Animal Ethics Procedures and Guidelines of the People’s Republic of China. The protocol was approved by the Institutional Animal Care and Use Committee (IACUC) of Zhejiang Academy of Medical Sciences.

## Author Contributions

BZ, SL, XZ, HD, DL, and QK developed the study protocol. BZ, JD, QT, and DL did the experiments. BZ analyzed the data and wrote the manuscript. SL revised the report. All authors read and approved the final manuscript.

## Conflict of Interest Statement

The authors declare that the research was conducted in the absence of any commercial or financial relationships that could be construed as a potential conflict of interest.
